# Using Aerial Thermal Imagery to Evaluate Water Status in *Vitis vinifera cv.* Loureiro

**DOI:** 10.3390/s22208056

**Published:** 2022-10-21

**Authors:** Cláudio Araújo-Paredes, Fernando Portela, Susana Mendes, M. Isabel Valín

**Affiliations:** 1 PROMETHEUS, Research Unit in Materials, Energy and Environment for Sustainability, Escola Superior Agrária, Instituto Politécnico de Viana do Castelo, Rua Escola Industrial e Comercial de Nun’Álvares, 4900-347 Viana do Castelo, Portugal; 2 Escola Superior Agrária, Instituto Politécnico de Viana do Castelo, 4900-347 Viana do Castelo, Portugal; 3 Centre for Research and Development in Agrifood Systems and Sustainability, Escola Superior Agrária, Instituto Politécnico de Viana do Castelo, 4900-347 Viana do Castelo, Portugal

**Keywords:** precision viticulture, unmanned aerial vehicle, thermal image, crop water stress index

## Abstract

The crop water stress index (CWSI) is a widely used analytical tool based on portable thermography. This method can be useful in replacing the traditional stem water potential method obtained with a Scholander chamber (PMS Model 600) because the latter is not feasible for large-scale studies due to the time involved and the fact that it is invasive and can cause damage to the plant. The present work had three objectives: (i) to understand if CWSI estimated using an aerial sensor can estimate the water status of the plant; (ii) to compare CWSI from aerial-thermographic and portable thermal cameras with stem water potential; (iii) to estimate the capacity of an unmanned aerial vehicle (UAV) to calculate and spatialize CWSI. Monitoring of CWSI (CWSI_P_) using a portable device was performed directly in the canopy, by measuring reference temperatures (T_dry_, T_wet,_ and canopy temperature (T_c_)). Aerial CWSI calculation was performed using two models: (i) a simplified CWSI model (CWSI_S_), where the T_dry_ and T_wet_ were estimated as the average of 1% of the extreme temperature, and (ii) an air temperature model (CWSI_Tair_) where air temperatures (T_air_ + 7 °C) were recorded as T_dry_ and in the T_wet_, considering the average of the lowest 33% of histogram values. In these two models, the T_c_ value corresponded to the temperature value in each pixel of the aerial thermal image. The results show that it was possible to estimate CWSI by calculating canopy temperatures and spatializing CWSI using aerial thermography. Of the two models, it was found that for CWSI_Tair_, CWSI_S_ (R^2^ = 0.55) evaluated crop water stress better than stem water potential. The CWSI_S_ had good correlation compared with the portable sensor (R^2^ = 0.58), and its application in field measurements is possible.

## 1. Introduction

In recent years, increased frequency of extremely high temperatures and low rainfall [1], together with the intensification of agricultural practices, has required that more attention be paid to the use of natural resources [2]. According to the most unfavorable scenario forecast by the Intergovernmental Panel Climate Change (IPCC) related to the representative concentration pathway (RCP) 8.5, water availability will decrease throughout the north of Portugal, and approximately 20% of the Vinho Verde Region, accompanied by a gradual increase in temperature [3]. In this scenario, grape quality and wine production [4,5] and the overall cycle of vines [6,7] will be affected by low soil water availability [3,5,6]. Strategies to achieve the full potential of a vineyard should include frequent monitoring of vine water status [7] and estimates of cultural evapotranspiration [8,9,10,11] to adopt the best irrigation practices.

It was shown in previous studies that several crop-based indices such as canopy temperature [12,13,14,15,16,17,18,19], chlorophyll content [20,21,22], and vegetation indices [23,24,25,26,27,28], can be used to determine crop water content, and have been employed for managing water in irrigated agriculture [29,30,31,32,33]. Canopy temperature [34] thermal indices obtained with ground-based or aerial-based sensors, can indicate crop transpiration and water stress, allowing the development of indices that assess vine water status [13,35] in a non-destructive and non-invasive way [18,36].

Unmanned aerial vehicles (UAVs), and thermal sensors coupled to UAVs, have been tested in precision viticulture [37,38] and offer advantages over measurements with ground-based (portable) sensors [13,35,39,40], due to their performance, flexibility of use, low operating cost, and very high spatial resolution [41,42]. Characterization of the spatial variability of crop water requirements is a prerequisite to apply precision scheduling strategies within orchards, and to enhance efficient water utilization to maintain vineyard yields and grape quality [36,37,43,44,45]. The development of models to spatialize data [18,42,46,47], converting localized information to continuous information, has changed the manner of observations from horizontal to vertical.

One of the most used thermal indices is the Crop Water Stress Index (CWSI), which varies between 0 (no stress) and 1 (maximum stress). Initially developed by [12], and later by [13], CWSI is based on a linear relationship between the difference in canopy temperature (Tc), air temperature, and baseline parameters such as dry leaf temperature (T_dry_), and wet leaf temperature (T_wet_) [13,35]. The CWSI method has been widely used in irrigated crops, and is considered a standard method. Many studies have been carried out in different crops, including nectarines [17,19], soybean [48], cotton [49], tomato [50], olive trees [51] and vines [52,53,54].

Methods have been developed methods to obtain T_dry_ and T_wet_ from aerial thermal images for calculating the CWSI. Of these, the methods that extract temperatures from the image histogram stand out because they simplify and streamline the process of obtaining data [49,55,56]. Using probability models to calculate the two temperatures (T_dry_ and T_wet_) for nectarines, [17] it was found that the temperature distribution can vary between different varieties and on the distribution of trees, even under similar irrigation levels. In cotton, extremes values of the surface temperature histogram were extracted to determine T_wet_ and T_dry_, demonstrating that the method was feasible to estimate the water status [49]. In vineyards, thermal imaging has shown that it can estimate water status accurately, with continuous representation, by the spatialization of data [18].

These methods have used a statistical approach to simplify the calculation of T_dry_ and T_wet_, taking these values as averages from the extremes values of the histogram [56,57]. The methods generally estimate better T_wet_ and T_dry_ values from the average temperatures of the lowest (coldest) and highest (hottest) part of the canopy temperature histogram than critical temperature values [49]. However, in non-continuous crops, such as vines, problems with the methods are increased because crop cover is not homogeneous (vineyard and soil), making it difficult to extract pure pixel values from the canopy, resulting in a bimodal histogram [58], which makes index spatialization difficult.

The present work aimed to evaluate the feasibility of using aerial thermal images to assess vine water status from CWSI spatialization in *Vitis vinifera cv*. Loureiro. Simplified CWSI (CWSI_S_) and CWSI air temperature (CWSI_Tair_), were used to compare data spatialization and to develop a structural flowchart. Model validation was attempted and analyses conducted concerning the variability of stem water potential Ψst (MPa) and CWSI_P_ (CWSI portable).

## 2. Materials and Methods

### 2.1. Study Area and Experimental Design

**Figure 1:**
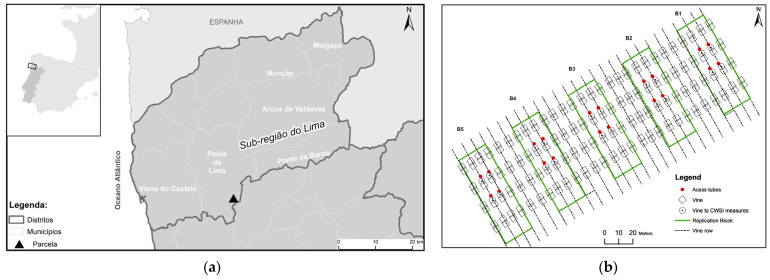
(**a**) Plot location in the northwest of Portugal. (**b**) Experimental design with five blocks (B1, B2, B3, B4 and B5) consisting of four rows with seven vines.

This work was carried out in 2021 in a commercial vineyard of *cv.* Loureiro of 5.5 ha in the Vinhos Verdes Region in the northwest of Portugal (41°40′32.2″ N; 8°32′05.9″ W; 175 elevation) (Figure 1a). The climate, characterized as Csb according to the Köppen–Geiger classification [59], has an Atlantic influence with moderate temperatures and thermal amplitudes and high rainfall (1200–1500 mm) concentrated in the winter months. The soil has a loamy texture (50% sand, 31% silt and 19% clay), an average depth of 1 m, organic matter content of 2.65% and a pH of 5.4. The soil water characteristics, obtained by laboratory methods, are a field capacity of 0.231 m^3^·m^−3^ (pF 2; 10 kPa) and a wilting coefficient of 0.121 m^3^·m^−3^ (pF 4.2; 1.55 MPa).

The vineyard was planted in 2001 in a north-south orientation, 1103 p rootstock, with 3.0 m between rows, and 2.0 m between vines (1666 plant/ha) trained to a single upward cordon. It has a drip irrigation system with one lateral per row (self-compensating drippers with 4 L h^−1^ flow rate).

The experimental design consisted of five blocks (B1, B2, B3, B4 and B5) of four rows of seven vines each measuring 1500 m^2^ located 50 m from the beginning of a row (Figure 1b). Only the vines of the two central lines were monitored, and this was conducted on three days of the year (DOY), i.e., day 182 (1 July), day 190 (9 July) and day 194 (13 July) for the following parameters: (i) soil water content (mm) using a capacitive probe (diviner); (ii) midday stem water potential (MPa) in four vines per block using a Scholander pressure chamber; (iii) the temperature of the canopy, using a portable thermal camera (FLIR e75, USA) in each of the 20 vines, on the three data recording dates for a total sample of 60 vines; and (iv) the temperature of the canopy using an aerial thermal camera (Zenmuse XT2, USA) supported by a UAV, (DJI Matrice 210, Frankfurt, Germany).

### 2.2. UAV Platform, Thermal Camera and Data Acquisition

**Figure 2:**
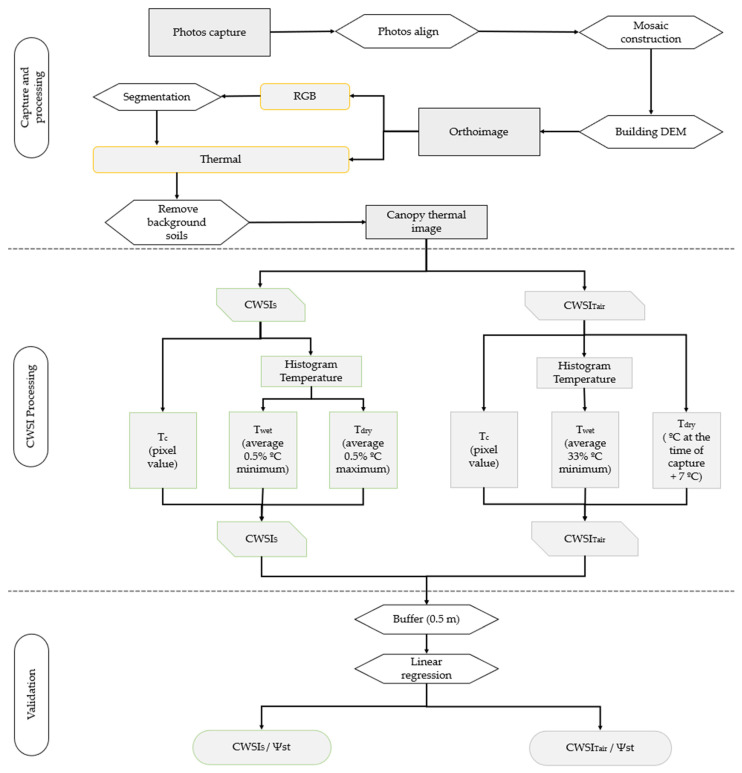
Flow diagram for calculating CWSI_S_ and CWSI_Tair_ and their spatialization.

The aerial images were collected using a DJI UAV, model Matrice 210, coupled with an aerial thermal camera [60,61] (Zenmuse XT2), with an 8 mm lens, resolution 640 × 512 pixels, frequency of 30 Hz, spectral range of 7.5–13.5 µm and a temperature range from −40 to 550 °C. The portable thermal camera (FLIR, e75) had a 17 mm lens, a resolution of 320 × 240 pixels, frequency of 30 Hz, spectral range of 7.5–14 µm and a temperature range from −20 to 120 °C. Sensors were used simultaneously. The images were taken at noon at the sampling site, and each pixel represented temperature in degrees Celsius. In the portable thermal camera, images were taken two meters from the canopy and perpendicular to the direction of the line. Aerial images were taken at a height of 60 m from groundwater potential, with a longitudinal and lateral overlap of 90% to obtain a photogrammetric mosaic (Figure 2). All images were acquired on clear days with minimal wind, and were evaluated on site. In the same location, a PMS Model 600 pressure camera was used to monitor stem water potential, because this is a sensitive indicator for vine water status and is frequently monitored to drive irrigation management.

### 2.3. Image Processing

Aerial thermal images analysed by Agisoft Metashape professional, Version 1.6.3 software to produce final orthophotos with a spatial resolution of 7 cm. To compare temperatures, the portable thermal camera was used to take images at the same time. Temperature calibration parameters such as emissivity, distance to a target, reflectivity temperature and relative humidity were entered into the Flir Tools, Version 5.13.18031.2002 software [62]. Production of the thermal and RGB orthophotography of the work area was carried out in eight main phases according to the flow diagram shown in Figure 2. To support georeferencing and geometric correction of the aerial image, a set of 16 points was assigned as control with a Root Mean Square Error (RMSE) less than 0.5. It was necessary to build a dense cloud of points by which, through photogrammetric restitution, it was possible to derive an extensive set of points that had information regarding an image’s latitude, longitude and altitude.

With this base, the texture of the image and the construction of a Digital Elevation Model (DEM) provided visual improvement and orthophotography production, by orthorectification, of the photogrammetric mosaic previously produced (Figure 2).

In the final phase, segmentation of the RGB aerial image was carried out using IDRISI software. The objective was to differentiate and isolate the areas between the rows, which were occupied by herbaceous vegetation, from areas occupied by vines so that they could be processed separately. Soil area extraction was necessary for the CWSI calculation to be carried out only within the vineyard area. For this procedure, and to delimit the segmentation polygons, a similarity of about 40% between the pixels was considered for analysis (Figure 2).

### 2.4. Calculation of Portable and Aerial CWSI

The calculation of the CWSI was based on the equation proposed by [13] and modified by [35] (Equation (1):

(1) $$\mathrm{CWSI}=\frac{\left(T_{c}-T_{\mathrm{wet}}\right)}{\left(T_{\mathrm{dry}}-T_{\mathrm{wet}}\right)}$$

where T_c_ is the canopy temperature obtained from the thermal image, and T_dry_ and T_wet_ are the reference temperatures (°C).

For the portable CWSI (CWSI_P_) the average T_c_ and average T_wet_ and T_dry_ were calculated using Flir Tools software. The reference temperatures (T_wet_ and T_dry_; °C) were obtained by selecting two healthy leaves close to each other in the canopy. Vaseline was applied to both sides of the T_dry_ leaf for 30 min before taking the readings to force the stomata to close, thereby preventing evapotranspiration, leading to a consequent increase in leaf temperature. For T_wet_, the leaf was sprayed with water, two minutes before taking readings to simulate maximum evapotranspiration rate [13,35].

**Figure 3:**
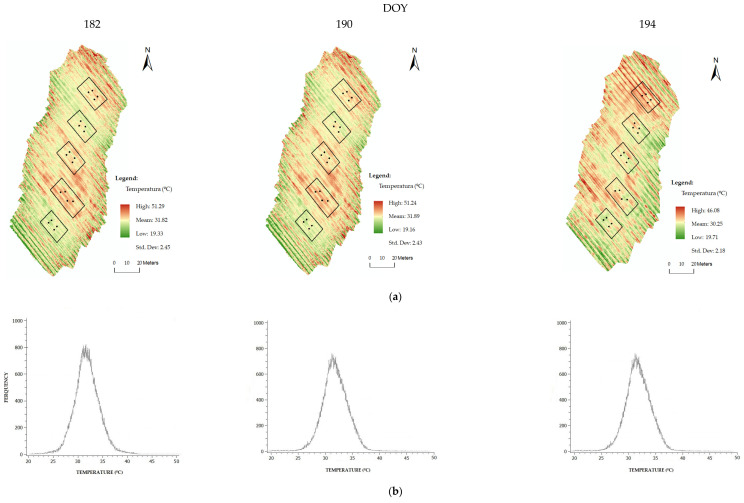
(**a**) Study area thermal orthophoto on different DOY. (**b**) Distribution temperature histogram relative to the soil and vineyard.

CWSI obtained from the aerial thermal sensor was processed by two methods. The first was CWSI_S_ based on the equation. The reference temperatures were obtained from the image histogram (Figure 3b), in which 99% of the values were assumed to correspond to normal temperatures and the remaining 1% to represent extremes, as discussed by [49], assuming that the T_dry_ represented the maximum temperature and T_wet_ the minimum temperature [49].

The air temperature method (CWSI_Tair_) was also based on Equation (1) but differed in the method for obtaining temperatures. T_c_ was the value of each pixel of the canopy temperature, T_wet_ was calculated from the average of 33% of the minimum temperatures within the histogram (Figure 3b), and T_dry_ was obtained from the air temperature plus 7 °C [55,63,64,65] (Figure 2).

### 2.5. Soil Water Content

The water content in the soil (φ) was measured at different depths (from 10 to 80 cm) by a capacitive probe to determine if soil water availability influenced the water status of the crop.

### 2.6. CWSI Validation with Stem Water Potential

To analyze the ability of the aerial CWSI to estimate the water status of the vine, coefficients of determination (R^2^) were calculated for each DOY and the data from the different CWSI (portable, simplified and air temperature), stem water potential (MPa) and CWSI_P_ with CWSI (simplified and air temperature). To validate the CWSI from the aerial sensor, georeferencing of the vines in each block was carried out. At these sampling sites, a 50 cm buffer was created to obtain the average value of the interior CWSI for later correlation with the field sample.

## 3. Results

### 3.1. Temperature Variation in the Sample Blocks

Table 1 shows the temperatures recorded in the five blocks on the three sampling dates by aerial thermal imaging. The wide canopy temperature range (26–30 °C) on all data collection days, highlighted the variability within each block and the ability of infrared thermography to support analysis and temperature variation from the vine canopy. All data followed a normal distribution and had a homogeneous dispersion on the different dates. In practically all cases, the median and average temperatures overlapped. On different DOY, the temperatures captured by the aerial thermal camera relative to the canopy and the ground varied between 19.2 and 51.3 °C. DOY 182 and 190 showed an overall variation of 32 °C (19.3–51.3 °C) with an average of 31.9 °C and a standard deviation of 2.5 and 2.4 °C, respectively. DOY 194 showed an overall variation of 26.4 °C (19.7–46.1 °C) with an average of 30.3 °C and a standard deviation of 2.2 °C.

From the temperature map (Figure 3a), on DOY 182 and 190 the temperatures inside each block were very close. Block 4 (B4) had the highest and block 5 (B5) had the lowest temperature. On DOY 194, block 1 (B1) had the highest and B5 the lowest temperature.

### 3.2. Calculation and Spatial Representation of Crop Water Stress Index (CWSI)

**Figure 4:**
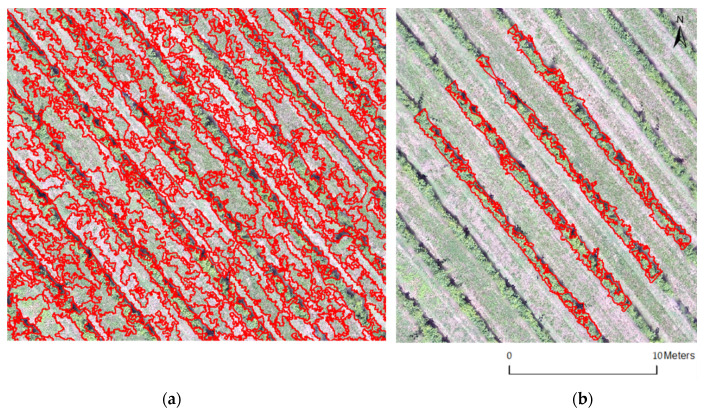
(**a**) Maps with the segmentation image. (**b**) Vine green vegetation considered for calculating canopy temperature.

For this process, image segmentation was used, so that only the temperature affected by the vine canopy was used. It was based on this new image that the CWSI was processed (Figure 4).

To calculate the CWSI (CWSI_S_ and CWSI_Tair_), different reference values were needed, depending on the method, to identify the difference in T_wet_ and T_dry_ values (Table 2). The T_wet_ for the CWSI_S_ had values of 25.5, 26.1 and 25.5 °C for DOY 182, 190, 194, respectively, all of which were lower than the air temperature. The CWSI_Tair_, presented values of 28.9, 29.0 and 27.8 °C, all of which were higher than the air temperature. The CWSI_S_ method resulted in lower T_wet_ values compared to CWSI_Tair_, which may have been due to the lower percentage of the histogram used in the first method. The T_dry_ for the CWSI_S_ had values of 38.2, 38.4 and 36.0 °C, and for the CWSI_Tair_ these were 35, 35 and 33 °C. The CWSI_S_ method resulted in higher T_dry_ values compared to the CWSI_Tair_ method, probably because the air temperature method, by normalizing this indicator, made it more stable than in the simplified method, which depended on temperature variability existing within and between the blocks.

**Figure 5:**
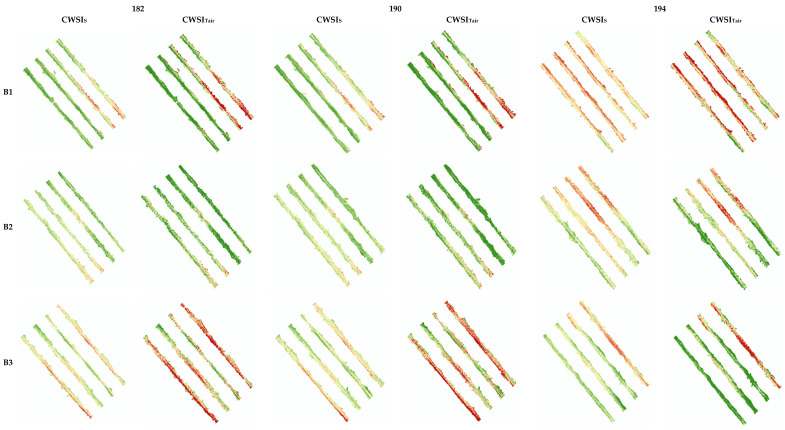

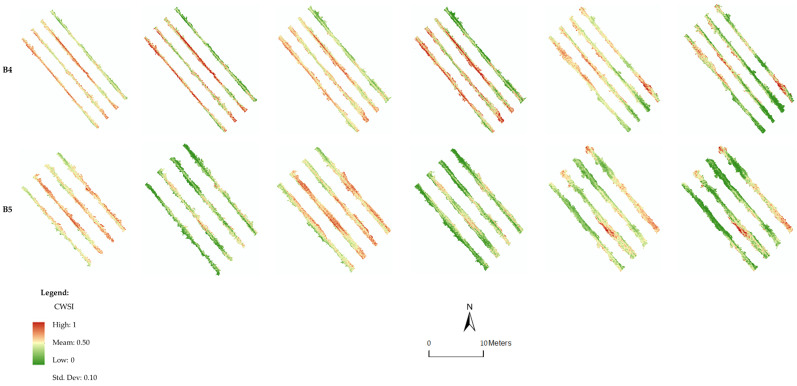
Maps of the five blocks of the CWSI_S_ and CWSI_Tair_ by DOY.

After obtaining the reference temperatures necessary to calculate CWSI, a spatial representation of the index was elaborated along the five blocks, where each pixel of the new image had its associated CWSI value, to determine if there was variability within and between blocks (Figure 5). The values for the two methods showed a range between 0 and 1 over the three DOY: the higher the value, the greater the water stress. CWSIs visually and numerically smoothed the data, whereas the CWSI_Tair_, by normalizing T_dry_, shifted the values close to the extremes. In general, it appears that the values of the two CWSI_S_ increased over the DOY, with a greater emphasis on the first block. It was also possible to verify a variation in CWSI within the sample block by changing the values represented, which may indicate plants with greater water stress in some areas.

The CWSI_P_ values of the sampling point, calculated for validation, varied in amplitude over the three dates from 0.36 to 0.70. The averages increased over the reading days, decreasing the CWSI_P_ amplitude. The same happened with the aerial sensors: the CWSI_S_ had an overall amplitude between 0.35 and 0.78, and the average values of the CWSI increased while the amplitude decreased. For the CWSI_Tair_, the amplitude increased from 0.45 to 0.88, while the average values of the CWSI increased from 0.57 to 0.72, decreasing its amplitude (Table 3).

The portable and aerial CWSI values remained close among the three methods. There was a slight increase from DOY 182 to DOY 194, which was explained by the lower soil water content in the latter, confirmed by a slight increase in stem water potential (Table 3). It should be noted that the CWSI_Tair_ had a very large coefficient of variation (0.42 to 0.44), whereas that of the CWSI_s_, on average, was less than half.

The soil water content varied between 110 and 199 mm for DOY 194 and DOY 182, respectively. The averages were 165, 159 and 144 mm for DOY 182, 190 and 194, respectively. All had low coefficients of variation (0.01–0.10). Thus, the water available in the soil decreased by about 20 mm overall (Table 4).

### 3.3. Correlation between Portable CWSI, Aerial CWSI and Stem Water Potential

To evaluate the relation between the proximal and remotely sensed water stress measurements with the vine water status, we determined the correlation between all the CWSI values (CWSI_Tair_, CWSI_S_ and CWSI_P_) and the stem water potential (Ψ_st_).

For each studied DOY (182, 190 and 194) the CWSI_P_ had the highest correlation, and the CWSI_Tair_ had the lowest correlation, with the Ψ_st_ (Table 5). These results are consistent with the global correlations, R^2^ = 0.59 for the CWSI_P_ and R^2^ = 0.49 for the CWSI_Tair_.

When we compared the CWSI, the highest correlation was obtained between CWSI_S_ and CWSI_P_ (R^2^ = 0.58) (Table 5). The fact that there were no changes in the determination coefficients when the UAV-supported thermal CWSI was incorporated (CWSI_Tair_ and CWSI_S_) suggested that the spatial structure represented the pattern of the stem water potential estimated from the simplified CWSI model.

## 4. Discussion

This study focused on the evaluation of thermal information to estimate the water status of a vineyard based on aerial and portable thermal images of the vine canopy. The method was fast and non-invasive compared to traditional methods such as using a pressure chamber to determine stem water potential. Using aerial thermal images captured with a UAV it was possible to produce an orthophotograph that comprised the temperature values of each pixel of the canopy. Segmentation of aerial RGB images isolated the canopy, discarded the area between the lines, facilitated calculation, and reduced thermal amplitudes that can influence the CWSI calculation model [16,17,49,55].

From analyzing the thermal images of three flights, it was possible to predict the water status of the plant (CWSI), taking into account the coefficient of determination when correlated with the stem water potential. Likewise, a moderate coefficient of determination was observed with the portable CWSI; however, this model did not allow a continuous surface of values. The results were in line with previous studies [17,49,55], but adding the ability to spatialize data, with the advantage of obtaining information from a vertical rather than a horizontal view.

Analyses of three days of capturing and processing data from portable thermal cameras and calculating respective CWSI_P_ concerning the stem water potential (R^2^ = 0.59) showed a moderate correlation, which agreed with the work developed by Garcia-Tejero et al. [35]. The results for each day, DOY 182 (R^2^ = 0.69), DOY 190 (R^2^ = 0.60) and DOY 194 (R^2^ = 0.58) had moderate correlations, which enhanced estimates of plant water status from the CWSI_P_. According to [41], the variability observed between the stem water potential and the CWSI for different DOY can be affected by parameters related to the phenological phase of the crop. The CWSI_P_ method, like use of the Scholander pressure chamber, is very expensive for determining an overall distribution because each reading only provides values for each plant in isolation.

Our results showed was possible to obtain crop water stress indices, calculated using date from two methods of aerial thermography, that made it possible to estimate and interpret the water status of the plant. Therefore, the method is as accurate as the Scholander pressure chamber method.

Given that it only needs to monitor air temperature during UAV flight, the calculation of the CWSI_Tair_ is a quick method for obtaining thermal data and, consequently, calculation of the CWSI. With this method, a moderate coefficient of determination was obtained with the stem water potential (R^2^ = 0.49). It was observed that DOY 182 (R^2^ = 0.49), DOY 190 (R^2^ = 0.53) and DOY (R^2^ = 0.52), which had moderate correlations, were in agreement with the results obtained by [55].

The CWSI_S_ calculation method required the least amount of time because it did not require field data. The correlation between the CWSI_S_ and stem water potential (R^2^ = 0.55) showed a moderate correlation, which was in line with the findings of [16,49]. When the analysis was aggregated by days, DOY 182 (R^2^ = 0.60), DOY 190 (R^2^ = 0.59) and DOY (R^2^ = 0.54) had moderate correlations and with approximations superior to that of CWSI_Tair_.

## 5. Conclusions

In this work, the development of two CWSI spatialization methods was explored, and the results were compared with those obtained by measuring stem water potential. It was found that CWSI_S_ could potentially estimate the water status of the vine using the reference temperatures (T_wet_ and T_dry_) from a histogram of image temperatures after segmentation.

The ability to obtain and spatialize thermal data has become extremely important, given that it is possible to image the plot as a whole, not just isolated plants. Furthermore, it is a non-invasive method, which allows taking a large number of measurements without weakening the plant in its phenological cycle. It also permits the collection and analysis of data on a large scale, and in a shorter time, whereas the CWSI_S_ method more closely estimates water stress of the crop. The CWSI_P_ method produced viable results, which were very close to those of the CWSI_S_, but it did not allow spatialization of the data.

Vegetation cover has a great influence on soil water preservation and thermal regulation. Where there is no turfgrass on the plot, the probability of obtaining a bimodal histogram can influence the CWSI results when applying the CWSI_S_ or CWSI_Tair_ methods.

Future research should consider information focused on leaf area, phenological phase, variety and age.

## Figures and Tables

**Table 1:** Statistics (average, median and standard deviation) of temperature taken with an aerial camera, relative humidity (HR %) and air temperature (T_air_ °C) on the three data collection days of the year (DOY).

	Statistics (°C)					T_air_	HR
DOY	Average	Median	St. Dev	Min.	Max.	(°C)	%
182	31.9	31.8	2.5	19.3	51.3	28	60
190	31.9	31.8	2.4	19.2	51.2	28	55
194	30.3	30.2	2.2	19.7	46.1	26	56

**Table 2:** Different methods of calculating T_wet_ and T_dry_ by DOY.

CWSI Types	Methods	DOY	T_wet_ (°C)	T_dry_ (°C)
CWSI_Tair_	Canopy temperature histogram (33% lowest)	182	28.9	-
		190	29.0	-
		194	27.8	-
	T_Air_ + 7 °C	182	-	35.0
		190	-	35.0
		194	-	33.0
CWSI_S_	Canopy temperature histogram (1%) ^1^	182	25.5	38.2
		190	26.1	38.4
		194	25.5	36.0

^1^ Average of the lowest and highest 0.5% of values in the canopy temperature histogram.

**Table 3:** Statistics for CWSI portable (CWSI_P_), CWSI air temperature (CWSI_Tair_), CWSI simplified (CWSI_S_), and water potential (Ψst) for the three DOY.

DOY	Descriptive Statistics	CWSI_P_	CWSI_Tair_	CWSI_S_	Ψst (MPa)
182	minimum	0.36	0.45	0.35	−0.45
	maximum	0.70	0.88	0.78	−0.70
	average	0.54	0.60	0.50	−0.53
	CV	0.18	0.44	0.20	0.13
190	minimum	0.40	0.46	0.35	−0.40
	maximum	0.68	0.82	0.70	−0.60
	average	0.57	0.65	0.55	−0.51
	CV	0.16	0.43	0.18	0.12
194	minimum	0.42	0.57	0.44	−0.50
	maximum	0.68	0.78	0.67	−0.75
	average	0.61	0.72	0.59	−0.61
	CV	0.11	0.42	0.12	0.11

**Table 4:** Statistics for soil water content (θ; mm) for the three dates (DOY).

DOY	Descriptive Statistics	B1	B2	B3	B4	B5
182	minimum	153.39	162.71	157.84	149.50	134.77
	maximum	186.52	166.38	199.37	177.01	160.20
	average	171.45	164.60	171.63	163.28	147.48
	CV	0.07	0.01	0.09	0.06	0.07
190	minimum	147.87	154.56	153.78	141.76	126.87
	maximum	179.07	164.55	195.76	170.77	153.73
	average	161.50	159.63	166.24	156.23	140.30
	CV	0.07	0.03	0.10	0.07	0.08
194	minimum	132.54	137.22	143.56	126.53	110.72
	maximum	168.60	153.23	182.01	159.45	144.80
	average	146.93	147.31	154.64	142.87	127.76
	CV	0.09	0.05	0.10	0.08	0.10

**Table 5:** Determination coefficients (R^2^) of the different CWSI_S_ and the stem water potential (Ψst).

Correlation	Global *n* = 60 (*p* < 0.05)	DOY 182 *n* = 20 (*p* < 0.05)	DOY 190 *n* = 20 (*p* < 0.05)	DOY 194 *n* = 20 (*p* < 0.05)
CWSI_P_/(Ψst)	0.59 (y = −0.6734x + 0.2063)	0.69	0.60	0.58
CWSI_S/_(Ψst)	0.55 (y = −0.7x + 0.165)	0.60	0.59	0.54
CWSI_Tair/_Ψst	0.49 (y = −0.6182x + 0.3216)	0.49	0.53	0.52
CWSI_S/_CWSI_P_	0.58 (y = 0.6468x + 0.2198)	0.59	0.60	0.57
CWSI_Tair/_CWSI_P_	0.34 (y = 0.3406x + 0.3487)	0.30	0.35	0.34

## Data Availability

Not applicable.
